# Obesity, metabolic health, and mortality in adults: a nationwide population-based study in Korea

**DOI:** 10.1038/srep30329

**Published:** 2016-07-22

**Authors:** Hae Kyung Yang, Kyungdo Han, Hyuk-Sang Kwon, Yong-Moon Park, Jae-Hyoung Cho, Kun-Ho Yoon, Moo-Il Kang, Bong-Yun Cha, Seung-Hwan Lee

**Affiliations:** 1 Division of Endocrinology and Metabolism, Department of Internal Medicine, Seoul St. Mary’s Hospital, College of Medicine, The Catholic University of Korea, Seoul 06591, Korea; 2 Department of Medical Statistics, College of Medicine, The Catholic University of Korea, Seoul 06591, Korea; 3 Division of Endocrinology and Metabolism, Department of Internal Medicine, Yeouido St. Mary’s Hospital, College of Medicine, The Catholic University of Korea, Seoul 07345, Korea; 4 Epidemiology Branch, National Institute of Environmental Health Sciences, National Institute of Health, Department of Health and Human Services, Research Triangle Park, NC 27709, USA

## Abstract

BMI, metabolic health status, and their interactions should be considered for estimating mortality risk; however, the data are controversial and unknown in Asians. We aimed to investigate this issue in Korean population. Total 323175 adults were followed-up for 96 (60–120) (median [5–95%]) months in a nationwide population-based cohort study. Participants were classified as “obese” (O) or “non-obese” (NO) using a BMI cut-off of 25 kg/m^2^. People who developed ≥1 metabolic disease component (hypertension, diabetes, dyslipidaemia) in the index year were considered “metabolically unhealthy” (MU), while those with none were considered “metabolically healthy” (MH). The MUNO group had a significantly higher risk of all-cause (hazard ratio, 1.28 [95% CI, 1.21–1.35]) and cardiovascular (1.88 [1.63–2.16]) mortality, whereas the MHO group had a lower mortality risk (all-cause: 0.81 [0.74–0.88]), cardiovascular: 0.73 [0.57–0.95]), compared to the MHNO group. A similar pattern was noted for cancer and other-cause mortality. Metabolically unhealthy status was associated with higher risk of all-cause and cardiovascular mortality regardless of BMI levels, and there was a dose-response relationship between the number of incident metabolic diseases and mortality risk. In conclusion, poor metabolic health status contributed more to mortality than high BMI did, in Korean adults.

Body mass index (BMI) is the most widely used measure of obesity, and higher BMI has been linked to the development of various metabolic diseases and their underlying pathophysiologies that could lead to increased mortality. However, this association is not necessarily linear, and many studies suggest the existence of ‘obesity paradox’ with different ranges of optimal BMI associated to mortality^1^,^2^,^3^,^4^. Possible explanations for this between-study heterogeneity are differences in the characteristics of the study population, ethnicities, follow-up period or the use of different reference categories of BMI^3^,^4^,^5^. Metabolic health status or comorbidities could also be important confounding factors in this relationship^6^,^7^.

Recently, subgroups of obesity showing unexpected metabolic profiles that deviate from normal BMI-metabolic disturbance relationship have gained much interest^8^,^9^,^10^. Although consensus on the defining criteria is lacking, these subgroups are usually classified by BMI and the degree of insulin resistance or the number of metabolic abnormalities. Despite being categorised into the same BMI group, the metabolic conditions and clinical outcomes may differ across different metabolic health statuses. Some obese individuals exhibit lower degrees of insulin resistance and visceral adiposity and more favorable cardiovascular risk profiles despite their high BMI, and therefore are classified as having metabolically healthy obese (MHO) phenotype^11^,^12^. In contrast, people with metabolically unhealthy non-obese (MUNO) phenotype are characterised by impaired insulin sensitivity, higher levels of abdominal adiposity, blood pressure (BP) and oxidative stress, lower physical activity energy expenditure, more atherogenic lipid profiles, and unfavorable adipokine profiles^13^,^14^,^15^,^16^. Therefore, it is evident that both BMI and metabolic health, and their interactions, should be considered to estimate the risk of mortality.

Several studies showed that people in the MHO or MUNO group have different risks in terms of the incidence of type 2 diabetes, cardiovascular diseases, and mortality; however, the results are inconsistent^17^,^18^,^19^. MUNO individuals are generally known to have higher risk of all-cause or cardiovascular mortality compared with their metabolically healthy counterpart^20^,^21^,^22^,^23^,^24^. Whether the MHO individuals have a lower risk of death compared with their metabolically unhealthy counterpart is controversial^25^,^26^,^27^,^28^. Large-scale studies in Asians are scarce; therefore, we aimed to investigate the risk of mortality according to BMI and metabolic health status using a nationwide dataset of the Korean population with a long-term follow-up period.

## Methods

### Data source and study population

The National Health Insurance System (NHIS) comprises a complete set of health information pertaining to 50 million Koreans, which includes an eligibility database (age, sex, socioeconomic variables, type of eligibility, income level, etc.), a medical treatment database (based on the medical bills that were claimed by medical service providers for their medical expense claims), a health examination database (results of general health examinations and questionnaires on lifestyle and behavior) and a medical care institution database (types of medical care institutions, location, equipment and number of physicians)^29^,^30^,^31^. The source population for this system is the Health Insurance Review and Assessment (HIRA) service. Healthcare providers submit reports on medical services provided under the health insurance policies to the HIRA service for a review of the medical costs incurred. Therefore, the HIRA database contains all the insurance claims information of approximately 97.0% of the Korean population. For this study, we used a customised NHIS database that included about 2.2% of the Korean population^32^,^33^. Subjects were selected using stratified random sampling to ensure that the sample was representative of the entire population.

The year when subjects first participated in the health examination was considered as the index year. Of the 412263 adults (age ≥20 years) who underwent health examinations between the year 2003 and 2008, those with a BMI <18.5 kg/m^2^ (n = 16952) or subjects who died in the index year (n = 146) were excluded. In order to avoid confounding by preexisting diseases and minimise the possible effects of reverse causality, those who had a history of cardiovascular disease or cancer (n = 22207) and subjects who were considered as having diabetes, hypertension, or dyslipidaemia (n = 49783) based on their medical treatment and health examination database before the index year were also excluded. Ultimately, the study population consisted of 323175 subjects (Supplementary Fig. 1). This study population was followed from baseline to the date of death or until December 31, 2013, whichever came first. This study was approved by the Institutional Review Board of The Catholic University of Korea (No. KC15EISI0432). Anonymised and de-identified information was used for analyses, and therefore informed consent was not obtained.

### Measurements

BMI was calculated as the subject’s weight in kilograms divided by the square of the subject’s height in meters. The BMI cutoff of 25 kg/m^2^ was adopted to define obesity for the Asian population enrolled in our study. Family histories of hypertension, stroke, heart disease, diabetes and cancer in the first-degree relatives were obtained using a questionnaire. Subjects were categorised as non-smokers, ex-smokers, or current smokers, and as drinking alcohol 0, 1–2, or ≥3 times/week based on the information obtained using the questionnaire. Regular exercise was defined as strenuous physical activity that was performed for at least 20 min, and subjects were categorised as exercising 0, 1–4, ≥5 times/week. Income level was dichotomised at the lower 20%. Blood samples were drawn after an overnight fast and measured for serum levels of glucose and total cholesterol. Hospitals wherein these health examinations were performed were certified by the NHIS and subjected to regular quality control.

### Definition of metabolic health status and cause of death

Three metabolic disease components (diabetes, hypertension and dyslipidaemia) were used to define metabolic health status. The presence of diabetes was defined according to the following criteria; (1) at least one claim per year for the prescription of antidiabetic medication under International Classification of Disease, 10th Revision (ICD–10) codes E10–14, or (2) fasting glucose level ≥7 mmol/L (obtained from the health examination database). The presence of hypertension was defined according to the presence of at least one claim per year for the prescription of antihypertensive agent under ICD-10 codes I10–I15, or systolic/diastolic BP ≥140/90 mmHg. The presence of dyslipidaemia was defined according to the presence of at least one claim per year for the prescription of antihyperlipidemic agent under ICD-10 codes E78, or total cholesterol ≥6.21 mmol/L (obtained from the health examination database). Among subjects with BMI <25 kg/m^2^, those who developed ≥1 metabolic disease component in the index year were considered MUNO individuals, while those with none of the three metabolic disease components were considered metabolically healthy non-obese (MHNO) individuals. Similarly, among subjects with BMI ≥25 kg/m^2^, metabolically unhealthy obese (MUO) and MHO were categorised according to the presence or absence of newly developed metabolic disease components in the index year, respectively. This definition led to a higher proportion of metabolically healthy individuals among the obese population compared with previous reports^26^,^27^,^28^.

Cause of death was classified according to the diagnostic codes of the ICD-10. All-cause mortality (any codes) was categorised into cardiovascular mortality (I00-I99), cancer mortality (C00–C97), and other-cause mortality (codes other than I00–I99 and C00–C97). The most frequent cause of other-cause mortality was injury, poisoning and other external causes (S00-T98; n = 1384), diseases of the respiratory system (J00-J99; n = 408), senility and unknown causes (R54 and R99; n = 392), and diseases of the digestive system (K00-K93; n = 311).

### Statistical analyses

Data are expressed as means (SD), geometric means (95% CI), or percentages. The characteristics of the 4 groups according to their BMI and metabolic health status were compared using one-way analysis of variance or Chi-squared tests. Survival curves were constructed with Kaplan-Meier estimates and compared using the log-rank test. Hazard ratios (HRs) and 95% CI values of all-cause, cardiovascular, cancer, and other-cause mortality were analysed using Cox proportional hazards models among 4 groups using the MHNO group as a reference. The proportional hazards assumptions were evaluated by the logarithm of cumulative hazards function based on Kaplan-Meier estimates for each group. Multivariable-adjusted proportional hazards model were applied; age and sex were adjusted in model 1, and smoking, alcohol drinking, exercise, and income status were further adjusted in model 2. A sensitivity analysis was performed excluding deaths that occurred within 3 years of follow-up to account for the possibility of reverse causation^2^. Next, BMI levels were further classified into 4 groups (normal-weight, 18.5– < 23; overweight, 23– < 25; obese I, 25– < 30; and obese II ≥ 30 kg/m^2^), and the HRs for mortality were evaluated among 8 subgroups according to their metabolic health status and BMI category. Risks for mortality according to the number of metabolic disease components were evaluated using a group of subjects with none of the 3 metabolic disease components as a reference group. A linear relationship (*P* for trend) was tested using general linear model. Lastly, mortality risk was assessed, according to the presence of various combinations of metabolic disease components. There was significant interactions of obese status with the relationship between the number (*P* = 0.043) or combinations (*P* = 0.018) of metabolic disease components and mortality. Therefore, the effect modification by obese status in these associations was evaluated through the stratified analysis. A *P* value < 0.05 was considered significant. Statistical analyses were performed using SAS version 9.3 (SAS Institute Inc., Cary, NC, USA).

## Results

### Baseline characteristics according to BMI and metabolic health status

Of the 323175 participants, 172681 (53.4%), 56025 (17.3%), 53303 (16.5%) and 41166 (12.7%) subjects were classified into the MHNO, MUNO MHO, and MUO group, respectively (Table 1). Mean BMI was approximately 22 kg/m^2^ in the non-obese groups and approximately 27 kg/m^2^ in the obese groups. By definition, systolic and diastolic BP, fasting glucose, and total cholesterol levels were higher in the MUNO and MUO groups than in the metabolically healthy groups (*P* < 0.001). Distribution of age groups and sex, prevalence of family histories, smoking, alcohol drinking, exercise, and income status also differed among the 4 groups (*P* < 0.001). Among subjects in the MUNO group, 68.0% had incident hypertension, 13.3% had incident diabetes, and 35.0% had incident dyslipidaemia. Among subjects in the MUO group, 71.9% had incident hypertension, 13.6% had incident diabetes, and 38.6% had incident dyslipidaemia.

During 96 (60–120) [median (5–95%)] months of follow-up, a total of 7786 subjects (2.41%) died, including 1278 deaths from cardiovascular diseases and 3164 deaths from cancer. The incidence of all-cause, cardiovascular, cancer, and other-cause mortality differed among the 4 groups. Mortality rate was highest in the MUNO group followed by that in the MUO group and was lowest in the MHO group, regardless of the cause of death (Table 2, Supplementary Fig. 2).

### Mortality according to BMI and metabolic health status

Compared with the subjects in the MHNO group, those in the MUNO group demonstrated a HR (95% CI) of 1.29 (1.23, 1.36) for all-cause mortality, 1.87 (1.63, 2.13) for cardiovascular mortality, 1.12 (1.03, 1.22) for cancer mortality, and 1.29 (1.19, 1.40) for other-cause mortality after adjusting for age and sex. Further adjustment of smoking status, alcohol drinking, exercise, and income status did not attenuate these associations. In contrast, compared with the MHNO group, the MHO group demonstrated a significantly lower HR (95% CI) for all-cause (0.81 [0.74, 0.88]), cardiovascular (0.73 [0.57, 0.95]), cancer (0.88 [0.77, 0.99]) and other-cause mortality (0.76 [0.66, 0.86]), after adjusting for possible confounding factors. Subjects in the MUO group showed a significantly higher HR (95% CI) for cardiovascular mortality (1.59 [1.33, 1.88]) but a lower HR (95% CI) for other-cause mortality (0.89 [0.79, 0.99]) compared with the MHNO group (Table 2). A sensitivity analysis, excluding deaths that occurred within 3 years of follow-up, showed similar results except for cancer mortality in the MUNO group and cardiovascular mortality in the MHO group which lost statistical significance (Supplementary Table 1). The results were mostly reproduced in males (Supplementary Table 2), but the protective effect seen in the MHO group was not observed in females (Supplementary Table 3). Similar pattern was noted for all-cause mortality in both non-smokers and current smokers (data not shown). These data suggest that MUNO individuals have an approximately 30% higher risk of all-cause or other-cause mortality, a 88% higher risk of cardiovascular mortality, and a 12% higher risk of cancer mortality, whereas MHO individuals had an approximately 20% lower risk for all-cause and other cause mortality and a 27% lower risk for cardiovascular mortality compared to the MHNO individuals.

**Figure 1:**
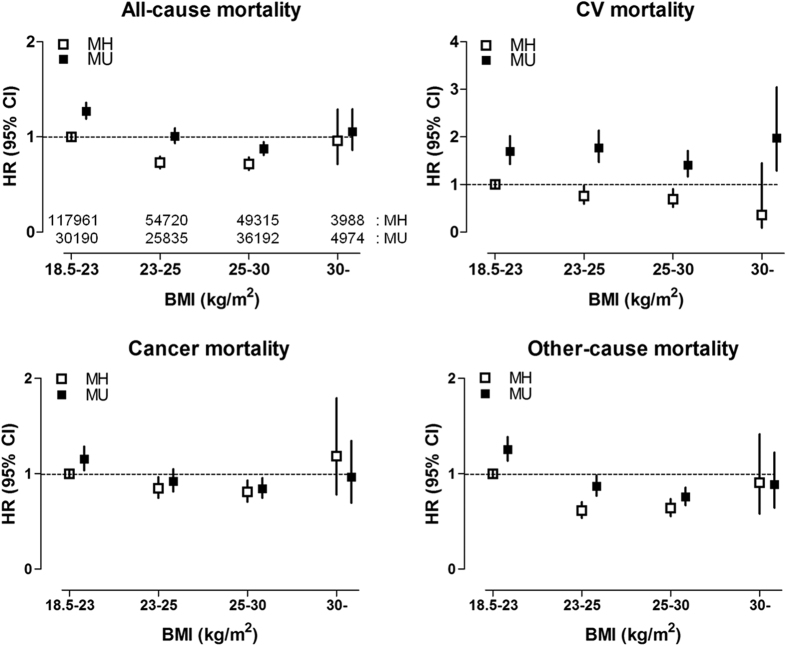
Mortality according to BMI and metabolic health status. Abbreviations: CV, cardiovascular; MH, metabolically healthy; MU, metabolically unhealthy. The HRs (95% CI) were calculated using a Cox proportional hazards model and are adjusted for age, sex, smoking, alcohol drinking, exercise, and income status. The numbers of subjects in each subgroup are described in the figure for all-cause mortality.

Next, the HRs for mortality were calculated among 8 subgroups according to their metabolic health status and 4 BMI categories (Fig. 1). Among metabolically healthy population, overweight and obese I group demonstrated significantly lower HRs for mortality compared to the normal-weight group. It was also notable that, compared to the metabolically healthy normal-weight group, subjects in the metabolically unhealthy normal-weight group demonstrated significantly higher HRs (95% CI) for all-cause (1.27 [1.19-1.36]), cardiovascular (1.70 [1.43, 2.02]), cancer (1.16 [1.04, 1.29]), and other-cause (1.26 [1.14, 1.39]) mortality. Same analysis was performed with the metabolically healthy group with a BMI 25– < 30 kg/m^2^ serving as the reference group, because this group showed the lowest HR for all-cause and cardiovascular mortality (Supplementary Fig. 3). Compared with the reference group, metabolically unhealthy status was associated with significantly higher HRs for all-cause and cardiovascular mortality regardless of the BMI categories. Subjects with a BMI 18.5– < 23 kg/m^2^ demonstrated significantly higher HRs for all-cause and cardiovascular mortality regardless of their metabolic health status, when compared to the reference group, suggesting the presence of ‘obesity paradox’ in this population. Cancer mortality and other-cause mortality were also significantly higher in both, metabolically healthy and unhealthy normal-weight group, compared to that in the reference group. These data suggest that metabolically unhealthy individuals are exposed to a higher risk of all-cause and cardiovascular mortality irrespective of their BMI status. In addition, normal-weight individuals have a higher mortality rate irrespective of their metabolic health status, compared with MHO individuals.

### Mortality according to the number or combination of metabolic diseases

Compared to those without any metabolic disease components, people with metabolic diseases showed a stepwise increase in the HRs across the number of components for all-cause, cardiovascular and other-cause mortality (Table 3). Individuals having all 3 components had a 77% higher risk for all-cause mortality and a 266% higher risk for cardiovascular mortality. The associations were augmented in non-obese subjects, but attenuated in obese subjects. The trend was similar between males and females (Supplementary Table 4).

**Figure 2:**
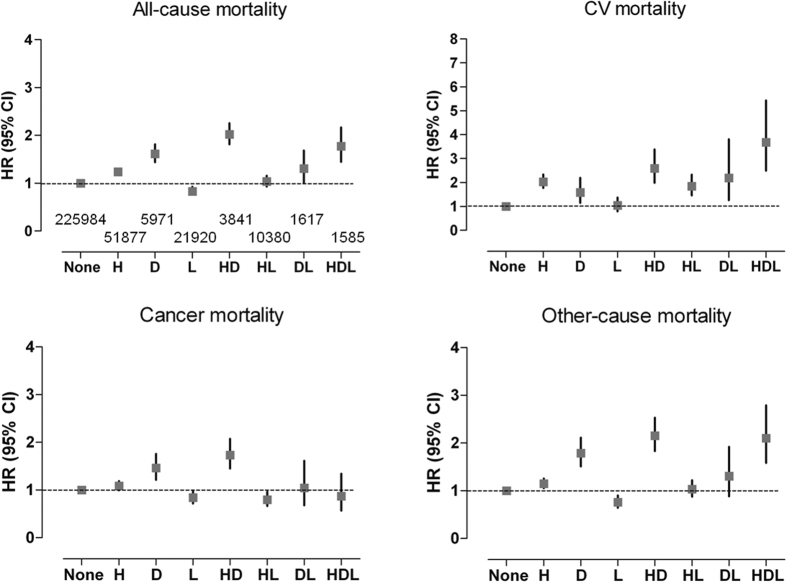
Mortality according to the combination of metabolic diseases. Abbreviations: CV, cardiovascular; H, hypertension; D, diabetes; L, dyslipidaemia. The HRs (95% CI) were calculated using a Cox proportional hazards model and are adjusted for age, sex, smoking, alcohol drinking, exercise, and income status. The numbers of subjects in each subgroup are described in the figure for all-cause mortality.

Next, we sought to examine the effect of various combinations of metabolic disease components on the HRs for mortality (Fig. 2). The development of hypertension and diabetes was associated with higher risk of all-cause, cardiovascular, and other-cause mortality. Subjects having only dyslipidaemia had lower risks of all-cause, cancer, and other-cause mortality, whereas this effect was abrogated by the combination of dyslipidaemia and other components. Therefore, subjects having both hypertension and diabetes demonstrated the highest HR for all-cause mortality. A significantly higher risk of cardiovascular death was noted in subjects with any combination with hypertension or diabetes. Cancer mortality was higher in subjects with diabetes or the combination of diabetes and hypertension, suggesting an important role of hyperglycaemia on malignancy-associated death. Generally, a similar pattern was noted in both non-obese and obese subjects (Supplementary Fig. 4).

## Discussion

From a large-scaled, nationwide, long-term follow-up study, we analysed the risk of mortality according to BMI and metabolic health status in the Korean population. First, individuals with the MUNO phenotype had a significantly higher risk of all-cause, cardiovascular, cancer, and other-cause mortality whereas individuals with the MHO phenotype had a significantly lower risk of death, compared with the MHNO group. Second, metabolically unhealthy status was associated with a higher risk of all-cause and cardiovascular mortality regardless of BMI levels, and there was a dose-response relationship between the number of incident metabolic diseases and the risk of mortality. Third, normal-weight individuals had a higher mortality rate, irrespective of their metabolic health status, compared with the MHO group. Finally, different combinations of metabolic disease components had different effects on the cause of mortality. Our data provide and expand the findings from other population-based studies, and suggest a meaningful influence of metabolic health status and their interaction with BMI on mortality.

Recently, several studies focused on the risk of mortality in the subgroups of obesity. In an analyses using the third National Health and Nutrition Examination Survey (NHANES III) in the US, subjects with a normal BMI but high body fat were shown to have a higher prevalence of cardiometabolic dysregulation and an increased risk for cardiovascular mortality compared to those with normal BMI and low body fat^20^,^21^. Another study using NHANES III data demonstrated a higher mortality in persons with normal weight and central obesity (defined by waist-hip ratio) than in persons with a similar BMI but no central obesity, or who were overweight or obese according to BMI only^23^. A community-based study performed in Scotland and England defined metabolic health status based on BP, high-density lipoprotein-cholesterol, diabetes, waist circumference, and C-reactive protein levels. Both non-obese and obese participants with at least two metabolic abnormalities, but not MHO individuals, were at an increased risk of all-cause and cardiovascular mortality over 7 years, compared with MHNO participants^27^. Similar findings were observed from a retrospective study of Korean subjects who participated in a health screening program. In this cohort, defining metabolic unhealthy status as having one or more metabolic syndrome components, MUO and MUNO groups were associated with higher cardiovascular mortality. However, significance was lost when subjects with prior diabetes, hypertension and cardiovascular disease were excluded, suggesting that these comorbid conditions explain much of the increased risk of mortality^7^. This contrasts with our data showing similar results after excluding subjects with known metabolic diseases and cardiovascular diseases. Different characteristics of study population, longer duration of follow-up and more numbers of events in our study might explain the discrepancy between two studies. A study involving elderly Korean people revealed the highest risk of death from all-cause and cardiovascular disease in normal-weight subjects with metabolic syndrome, which was significantly higher than that in MHO individuals. The lowest risk of death was seen in overweight subjects without metabolic syndrome^22^. A meta-analysis of 14 prospective cohort studies also confirmed that the individuals with MUNO or MUO phenotype have an increased risk of mortality^19^. Our data, suggesting an important role of metabolic health and a significantly higher mortality in MUNO subjects are generally in line with previous reports.

Whether individuals within the MHO group carry a lower risk of mortality than the MUO group is controversial. Ortega *et al.* considered metabolically healthy status when meeting 0 or 1 of the criteria for metabolic syndrome in a mostly Caucasian population, and suggested that MHO is a benign condition with a 30–50% lower risk of all-cause mortality and cardiovascular diseases than that in MUO individuals^28^. Other studies using various definitions for metabolic health have also reached similar conclusions^26^,^27^. However, a study that analysed NHANES III data argued that obesity, even in the absence of metabolic abnormalities (defined as insulin resistance by homeostasis model assessment or fulfillment of 2 or more metabolic syndrome criteria) is associated with an increased all-cause mortality similar to that in metabolically abnormal obese individuals^25^. It is also debated whether obesity itself increases the risk of mortality even in the absence of metabolic abnormalities, when compared to MHNO subjects. Some studies indicate that MHO individuals are not at a significantly increased risk of mortality compared to their normal-weight counterparts^26^,^27^,^28^, while others showed a 2- to 3-fold increased risk^24^,^25^. Our data suggest that the MHO group has significantly lower risk of death, approximately 20% (for all-cause and other cause), and 27% (for cardiovascular), when compared with the MHNO group. The differences in defining metabolic health might be an important factor leading to discrepant results^24^. In addition, Asians use different cut-off values for BMI and are known to have higher percentage of body fat than Caucasians with a similar BMI^34^, which is a notable factor in interpreting the results.

From various studies, BMI levels have been demonstrated to have U- or J-shaped risk relationships with mortality in both, Asian and Western populations^1^,^2^,^3^,^4^,^35^,^36^. In our study, among the participants without incident metabolic diseases, those with normal-weight demonstrated a higher risk for all-cause, cardiovascular, cancer, and other-cause mortality compared to those with moderate obesity. Metabolically unhealthy subjects with normal-weight also had the highest HR for all-cause mortality. Recently, Kim *et al.* reported that subjects with moderate obesity (BMI 25– < 30 kg/m^2^) had a lower risk of mortality compared to normal weight, underweight, and overweight subjects in the general Korean population^33^. These findings support the presence of the ‘obesity paradox’, which has been widely observed in different ethnic groups^37^,^38^. Obese subjects with incident or pre-existing hypertension, diabetes, heart failure, ischemic heart disease, or chronic kidney disease have demonstrated better survival compared to those with lower BMI levels^39^,^40^,^41^. Favorable fat mass/fat-free mass ratio, nutritional status, cardiorespiratory fitness, greater likelihood of receiving optimal medical treatment, and cardioprotective metabolic effects of increased body fat have been suggested to explain the protective effect of obesity^3^,^41^. However, others argue that the ‘obesity paradox’ does not exist and have cited selection or survival bias, treatment bias, unintentional weight loss from comorbidities, and other confounding variables as possible alternate explanations^42^.

As expected, a dose-response relationship between the number of incident metabolic diseases and the risk of mortality was observed. Having only one metabolic disease was associated with a significantly higher risk, and developing all three diseases augmented this effect. An interesting study by the Emerging Risk Factors Collaboration showed that associations of myocardial infarction, stroke, and diabetes with mortality are multiplicative^6^. HR for mortality was about 2 in patients having 1 condition; about 4 for a combination of any 2 conditions; and about 8 for a combination of all 3 conditions. Having established cardiovascular diseases or its equivalent (diabetes) might have led to higher HRs than those in our study, which accounted for the risk factors of cardiovascular diseases. In analysing the effect of various combinations of metabolic diseases on mortality, we found that hypertension was an essential factor associated with a higher risk of cardiovascular mortality. A pooled analysis of 97 cohorts investigating the role of metabolic mediators on the effect of BMI on coronary heart disease and stroke also emphasised that BP was the most important mediator^43^. In terms of cancer mortality, diabetes was the most relevant factor; this is supported by the well-known association between diabetes and cancer^44^. The lower risk of mortality in subjects having dyslipidaemia might be attributable to an increased chance of receiving statin therapy or appropriate risk management including lifestyle modification. However, in subjects with multiple metabolic disease components, the magnitude of metabolic deterioration may outweigh the benefit of risk management.

Our study has a strength that it is a large-scale, Asian study evaluating the influence of obesity and metabolic health on mortality, conducted using a nationwide dataset. For diagnostic accuracy, we used combinations of disease code and medication prescription data, as well as laboratory data from health examination database. Furthermore, to minimise reverse causality, we excluded participants with preexisting metabolic diseases, cardiovascular diseases, and malignancy. However, there are limitations in our study. First, because disease codes might not represent a participant’s exact disease status and prescription of medications does not guarantee compliance^30^, there might be errors in the classification of metabolic health status. Second, it is well known that a considerable number of subjects have changes in their BMI or metabolic health status over time, leading to different clinical outcomes^45^,^46^, but our analysis did not account for this possibility. Third, we used BMI to define obesity, while measurement of waist circumference might have provided a better correlation to visceral adiposity^47^. In addition, information on recent body weight change before enrollment was unavailable, which might have led to a misclassification of baseline risk status. Fourth, there were limited number of participants with morbid obesity; therefore, detailed analysis in this range was unavailable. Fifth, higher mortality in normal-weight individuals might be partly attributed to sarcopenia, but body composition was not measured in this cohort. Lastly, because approximately 40% of subjects in customised NHIS database participated in the health examination, our dataset is not a representative of general population and a possibility of healthy user bias should be considered.

In conclusion, we demonstrated different risks for mortality according to obesity (measured as BMI), metabolic health status, and their interactions in Korean adults using a nationwide population-based cohort. Individuals with the MUNO phenotype had an approximately 30% higher risk of all-cause mortality, whereas the MHO group had an approximately 20% lower mortality risk compared to the MHNO group. Further studies with consensus on the defining criteria of metabolic health are required to clearly delineate the effect of metabolic health status on mortality.

## Additional Information

**How to cite this article**: Yang, H. K. *et al.* Obesity, metabolic health, and mortality in adults: a nationwide population-based study in Korea. *Sci. Rep.* **6**, 30329; doi: 10.1038/srep30329 (2016).

## Supplementary Material

Supplementary Information

## Figures and Tables

**Table 1:** Characteristics of subjects according to body mass index and metabolic health status.

Variable	MHNO (n = 172681)	MUNO (n = 56025)	MHO (n = 53303)	MUO (n = 41166)
Age (years, %)				
20–39	49.4	23.1	42.2	28.5
40–64	46.3	62.0	53.8	62.2
≥65	4.3	14.9	4.0	9.3
Sex (male, %)	47.8	60.2	62.5	66.8
Height (cm)	164.0 (8.7)	163.1 (9.2)	165.2 (9.4)	164.6 (9.6)
Weight (kg)	59.2 (8.1)	60.3 (8.3)	74.0 (9.9)	74.7 (10.7)
Body mass index (kg/m^2^)	21.9 (1.7)	22.6 (1.6)	27.0 (2.0)	27.5 (2.3)
Systolic BP (mmHg)	115.4 (11.2)	134.3 (17.5)	120.0 (10.5)	137.2 (16.7)
Diastolic BP (mmHg)	72.2 (8.0)	84.8 (11.4)	75.0 (7.4)	86.6 (11.1)
Fasting glucose (mmol/L)	4.9 (0.6)	5.6 (2.1)	5.1 (0.7)	5.7 (1.9)
Total cholesterol (mmol/L)	4.7 (0.7)	5.5 (1.2)	4.9 (0.7)	5.7 (1.1)
Family history (%)				
Hypertension	7.8	8.7	8.1	10.1
Diabetes	7.7	6.9	9.0	8.9
Heart disease	2.7	2.6	3.0	2.9
Stroke	4.3	4.9	4.8	5.2
Cancer	12.6	12.0	13.0	12.2
Smoking (%)				
None	69.3	63.5	61.8	60.2
Ex-smoker	3.7	4.6	5.4	5.9
Current smoker	27.0	31.9	32.8	33.9
Alcohol drinking (%)				
None	49.3	49.2	45.8	44.8
1–2 times/wk	22.9	16.7	21.0	17.7
≥3 times/wk	27.8	34.1	33.2	37.5
Regular exercise (%)				
None	57.0	55.9	50.6	52.1
1–4 times/wk	36.6	35.4	41.7	39.5
≥5 times/wk	6.4	8.7	7.7	8.4
Income (lower 20%, %)	16.4	16.8	15.2	15.3
Hypertension (%)				
By code/medication	0	11.2	0	13.2
By health examination	0	56.8	0	58.7
Diabetes (%)				
By code/medication	0	3.3	0	3.2
By laboratory examination	0	10.0	0	10.4
Dyslipidaemia (%)				
By code/medication	0	5.9	0	6.9
By laboratory examination	0	29.1	0	31.7

Abbreviations: BP, blood pressure; MHNO, metabolically healthy non-obese; MHO, metabolically healthy obese; MUNO, metabolically unhealthy non-obese; MUO, metabolically unhealthy obese. Data are expressed as the means (SD), %, or geometric means (95% confidence interval).

**Table 2:** Mortality according to body mass index and metabolic health status.

	MHNO	MUNO	MHO	MUO
Person-year	1366032	454731	421845	335448
All-cause mortality				
n (%)	2861 (1.66)	3010 (5.37)	733 (1.38)	1182 (2.87)
Mortality rate/1000 person-year	2.09	6.62	1.74	3.52
Model 1	1 (ref)	1.29 (1.23, 1.36)	0.79 (0.73, 0.85)	0.96 (0.90, 1.03)
Model 2	1 (ref)	1.28 (1.21, 1.35)	0.81 (0.74, 0.88)	0.98 (0.91, 1.06)
Cardiovascular mortality				
n (%)	361 (0.21)	597 (1.07)	78 (0.15)	242 (0.59)
Mortality rate/1000 person-year	0.26	1.31	0.19	0.72
Model 1	1 (ref)	1.87 (1.63, 2.13)	0.69 (0.54, 0.89)	1.53 (1.30, 1.80)
Model 2	1 (ref)	1.88 (1.63, 2.16)	0.73 (0.57, 0.95)	1.59 (1.33, 1.88)
Cancer mortality				
n (%)	1233 (0.71)	1112 (1.98)	343 (0.64)	476 (1.16)
Mortality rate/1000 person-year	0.90	2.45	0.81	1.42
Model 1	1 (ref)	1.12 (1.03, 1.22)	0.85 (0.75, 0.96)	0.90 (0.81, 1.00)
Model 2	1 (ref)	1.12 (1.02, 1.22)	0.88 (0.77, 0.99)	0.90 (0.81, 1.01)
Other-cause mortality				
n (%)	1267 (0.73)	1301 (2.32)	312 (0.59)	464 (1.13)
Mortality rate/1000 person-year	0.93	2.86	0.74	1.38
Model 1	1 (ref)	1.29 (1.19, 1.40)	0.75 (0.66, 0.85)	0.86 (0.77, 0.95)
Model 2	1 (ref)	1.26 (1.16, 1.37)	0.76 (0.66, 0.86)	0.89 (0.79, 0.99)

Abbreviations: MHNO, metabolically healthy non-obese; MHO, metabolically healthy obese; MUNO, metabolically unhealthy non-obese; MUO, metabolically unhealthy obese.

Data are expressed as the HR (95% confidence interval).

Model 1: Adjusted for age and sex.

Model 2: Adjusted for Model 1 + smoking, alcohol drinking, exercise, and income.

**Table 3:** Mortality according to the number of metabolic diseases.

	All-cause mortality	Cardiovascular mortality	Cancer mortality	Other-cause mortality
Total subjects				
0 (n = 225984)	1 (ref)	1 (ref)	1 (ref)	1 (ref)
1 (n = 79768)	1.18 (1.12, 1.25)	1.80 (1.58, 2.05)	1.07 (0.98, 1.16)	1.12 (1.04, 1.21)
2 (n = 15838)	1.36 (1.26, 1.47)	2.09 (1.74, 2.51)	1.11 (0.97, 1.26)	1.40 (1.24, 1.58)
3 (n = 1585)	1.77 (1.44, 2.16)	3.66 (2.48, 5.41)	0.87 (0.57, 1.34)	2.09 (1.58, 2.78)
*P* for trend	<0.001	<0.001	0.130	<0.001
Body mass index <25 kg/m^2^				
0 (n = 172681)	1 (ref)	1 (ref)	1 (ref)	1 (ref)
1 (n = 47591)	1.21 (1.14, 1.29)	1.76 (1.52, 2.05)	1.10 (0.99, 1.20)	1.16 (1.06, 1.27)
2 (n = 7770)	1.45 (1.31, 1.59)	2.00 (1.60, 2.50)	1.17 (0.99, 1.38)	1.54 (1.34, 1.77)
3 (n = 664)	2.34 (1.84, 2.97)	4.84 (3.08, 7.61)	1.29 (0.78, 2.11)	2.57 (1.82, 3.63)
*P* for trend	<0.001	<0.001	0.011	<0.001
Body mass index ≥25 kg/m^2^				
0 (n = 53303)	1 (ref)	1 (ref)	1 (ref)	1 (ref)
1 (n = 32177)	1.20 (1.08, 1.34)	2.11 (1.59, 2.81)	1.05 (0.89, 1.23)	1.13 (0.96, 1.34)
2 (n = 8068)	1.38 (1.19, 1.59)	2.70 (1.91, 3.82)	1.08 (0.86, 1.36)	1.35 (1.08, 1.70)
3 (n = 921)	1.29 (0.89, 1.87)	2.68 (1.23, 5.84)	0.47 (0.19, 1.14)	1.85 (1.13, 3.02)
*P* for trend	<0.001	<0.001	0.991	0.001

Data are expressed as the HR (95% confidence interval).

Adjusted for age, sex, smoking, alcohol drinking, exercise, and income.
