# Supplementary material for: Obesity, metabolic health, and mortality in adults: a nationwide population-based study in Korea
Source: Sci Rep. 2016 Jul 22;6:30329. doi: 10.1038/srep30329 (PMC4957204; doi:10.1038/srep30329)

Supplementary Information

Obesity, metabolic health, and mortality in adults: a nationwide population-based study in Korea

Hae Kyung Yang, Kyungdo Han, Hyuk-Sang Kwon, Yong-Moon Park, Jae-Hyoung Cho, Kun-Ho Yoon, Moo-Il Kang, Bong-Yun Cha, Seung-Hwan Lee

Supplementary Tables

Supplementary Table 1. Mortality according to body mass index and metabolic health status (sensitivity analysis excluding deaths within 3 years of follow-up)

	MHNO	MUNO	MHO	MUO
Person-year	1364659	453339	421465	334910
All-cause mortality				
n (%)	1980 (1.15)	2115 (3.84)	505 (0.95)	850 (2.08)
Mortality rate/1000 person-year	1.45	4.67	1.20	2.54
Model 1	1 (ref)	1.29 (1.21, 1.37)	0.78 (0.71, 0.86)	0.98 (0.90, 1.06)
Model 2	1 (ref)	1.27 (1.19, 1.36)	0.81 (0.73, 0.89)	1.00 (0.92, 1.09)
Cardiovascular mortality				
n (%)	256 (0.15)	417 (0.76)	58 (0.11)	161 (0.39)
Mortality rate/1000 person-year	0.19	0.92	0.14	0.48
Model 1	1 (ref)	1.81 (1.54, 2.12)	0.73 (0.55, 0.97)	1.40 (1.15, 1.71)
Model 2	1 (ref)	1.82 (1.54, 2.15)	0.77 (0.57, 1.04)	1.48 (1.20, 1.82)
Cancer mortality				
n (%)	860 (0.50)	773 (1.40)	236 (0.44)	357 (0.87)
Mortality rate/1000 person-year	0.63	1.71	0.56	1.07
Model 1	1 (ref)	1.12 (1.01, 1.24)	0.83 (0.72, 0.96)	0.95 (0.84, 1.08)
Model 2	1 (ref)	1.09 (0.98, 1.21)	0.85 (0.73, 0.99)	0.95 (0.83, 1.08)
Other-cause mortality				
n (%)	864 (0.50)	925 (1.68)	211 (0.40)	332 (0.81)
Mortality rate/1000 person-year	0.63	2.04	0.50	0.99
Model 1	1 (ref)	1.30 (1.18, 1.42)	0.75 (0.64, 0.87)	0.87 (0.77, 0.99)
Model 2	1 (ref)	1.27 (1.15, 1.41)	0.77 (0.65, 0.90)	0.90 (0.79, 1.03)

Abbreviations: MHNO, metabolically healthy non-obese; MHO, metabolically healthy obese; MUNO, metabolically unhealthy non-obese; MUO, metabolically unhealthy obese. Data are expressed as the HR (95% confidence interval).

Model 1: Adjusted for age and sex.

Model 2: Adjusted for Model 1 + smoking, alcohol drinking, exercise, and income.

Supplementary Table 2. Mortality according to body mass index and metabolic health status (male)

	MHNO	MUNO	MHO	MUO
Person-year	666994	278310	267982	227848
All-cause mortality				
n (%)	2024 (2.45)	2154 (6.39)	474 (1.42)	811 (2.95)
Mortality rate/1000 person-year	2.94	7.50	1.73	3.48
Model 1	1 (ref)	1.29 (1.22, 1.38)	0.72 (0.65, 0.79)	0.94 (0.87, 1.02)
Model 2	1 (ref)	1.28 (1.20, 1.37)	0.74 (0.66, 0.82)	0.98 (0.90, 1.07)
Cardiovascular mortality				
n (%)	247 (0.30)	401 (1.19)	52 (0.16)	162 (0.59)
Mortality rate/1000 person-year	0.36	1.40	0.18	0.69
Model 1	1 (ref)	1.88 (1.60, 2.21)	0.67 (0.50, 0.91)	1.55 (1.27, 1.89)
Model 2	1 (ref)	1.92 (1.61, 2.27)	0.75 (0.55, 1.02)	1.63 (1.32, 2.02)
Cancer mortality				
n (%)	879 (1.06)	823 (2.44)	210 (0.63)	324 (1.18)
Mortality rate/1000 person-year	1.29	2.86	0.78	1.38
Model 1	1 (ref)	1.11 (1.01, 1.22)	0.75 (0.64, 0.87)	0.87 (0.76, 0.98)
Model 2	1 (ref)	1.11 (0.99, 1.23)	0.77 (0.66, 0.91)	0.88 (0.77, 1.01)
Other-cause mortality				
n (%)	898 (1.09)	930 (2.76)	212 (0.64)	325 (1.18)
Mortality rate/1000 person-year	1.29	3.24	0.77	1.40
Model 1	1 (ref)	1.31 (1.20, 1.44)	0.70 (0.60, 0.82)	0.85 (0.75, 0.96)
Model 2	1 (ref)	1.28 (1.15, 1.41)	0.71 (0.60, 0.83)	0.90 (0.79, 1.03)

Abbreviations: MHNO, metabolically healthy non-obese; MHO, metabolically healthy obese; MUNO, metabolically unhealthy non-obese; MUO, metabolically unhealthy obese. Data are expressed as the HR (95% confidence interval).

Model 1: Adjusted for age.

Model 2: Adjusted for Model 1 + smoking, alcohol drinking, exercise, and income.

Supplementary Table 3. Mortality according to body mass index and metabolic health status (female)

	MHNO	MUNO	MHO	MUO
Person-year	699037	176420	153863	107600
All-cause mortality				
n (%)	837 (0.93)	856 (3.84)	259 (1.30)	371 (2.72)
Mortality rate/1000 person-year	1.17	4.75	1.66	3.33
Model 1	1 (ref)	1.27 (1.15, 1.40)	0.93 (0.81, 1.06)	0.97 (0.86, 1.10)
Model 2	1 (ref)	1.27 (1.14, 1.40)	0.94 (0.81, 1.09)	0.97 (0.85, 1.10)
Cardiovascular mortality				
n (%)	114 (0.13)	196 (0.88)	26 (0.13)	80 (0.59)
Mortality rate/1000 person-year	0.16	1.09	0.16	0.72
Model 1	1 (ref)	1.74 (1.37, 2.20)	0.69 (0.45, 1.06)	1.36 (1.02, 1.81)
Model 2	1 (ref)	1.74 (1.37, 2.22)	0.67 (0.43, 1.05)	1.40 (1.04, 1.88)
Cancer mortality				
n (%)	354 (0.39)	289 (1.30)	133 (0.67)	152 (1.11)
Mortality rate/1000 person-year	0.49	1.59	0.86	1.41
Model 1	1 (ref)	1.21 (1.03, 1.42)	1.15 (0.94, 1.41)	1.08 (0.89, 1.31)
Model 2	1 (ref)	1.19 (1.01, 1.41)	1.17 (0.95, 1.44)	1.05 (0.86, 1.29)
Other-cause mortality				
n (%)	369 (0.41)	371 (1.66)	100 (0.50)	139 (1.02)
Mortality rate/1000 person-year	0.52	2.06	0.64	1.20
Model 1	1 (ref)	1.17 (1.01, 1.36)	0.81 (0.65, 1.01)	0.79 (0.65, 0.96)
Model 2	1 (ref)	1.17 (1.00, 1.37)	0.82 (0.65, 1.04)	0.79 (0.64, 0.97)

Abbreviations: MHNO, metabolically healthy non-obese; MHO, metabolically healthy obese; MUNO, metabolically unhealthy non-obese; MUO, metabolically unhealthy obese. Data are expressed as the HR (95% confidence interval).

Model 1: Adjusted for age.

Model 2: Adjusted for Model 1 + smoking, alcohol drinking, exercise, and income.

Supplementary Table 4. Mortality according to the number of metabolic diseases in male and female

	All-cause mortality	Cardiovascular mortality	Cancer mortality	Other-cause mortality
Male				
0 (n = 115883)	1 (ref)	1 (ref)	1 (ref)	1 (ref)
1 (n = 50289)	1.20 (1.13, 1.28)	1.83 (1.56, 2.15)	1.07 (0.97, 1.18)	1.15 (1.05, 1.26)
2 (n = 9913)	1.44 (1.31, 1.59)	2.19 (1.74, 2.75)	1.18 (1.01, 1.38)	1.51 (1.31, 1.74)
3 (n = 1042)	1.69 (1.31, 2.18)	3.56 (2.15, 5.90)	0.94 (0.57, 1.57)	1.91 (1.33, 2.75)
P for trend	< 0.001	< 0.001	0.057	< 0.001
Female				
0 (n = 110101)	1 (ref)	1 (ref)	1 (ref)	1 (ref)
1 (n = 29479)	1.15 (1.05, 1.26)	1.69 (1.35, 2.13)	1.11 (0.95, 1.28)	1.03 (0.89, 1.19)
2 (n = 5925)	1.20 (1.04, 1.38)	1.83 (1.35, 2.48)	1.06 (0.83, 1.34)	1.14 (0.92, 1.41)
3 (n = 543)	1.88 (1.35, 2.62)	3.48 (1.88, 6.46)	0.81 (0.36, 1.82)	2.26 (1.44, 3.55)
P for trend	< 0.001	< 0.001	0.466	0.027

Data are expressed as the HR (95% confidence interval).

Adjusted for age, sex, smoking, alcohol drinking, exercise, and income.

Supplementary Figures

Supplementary Figure 1. Flow diagram of study subjects and their follow-up

Abbreviations: BMI, body mass index; CVD, cardiovascular disease; DM, diabetes mellitus; HTN, hypertension; MHNO, metabolically healthy non-obese; MHO, metabolically healthy obese; MUNO, metabolically unhealthy non-obese; MUO, metabolically unhealthy obese.

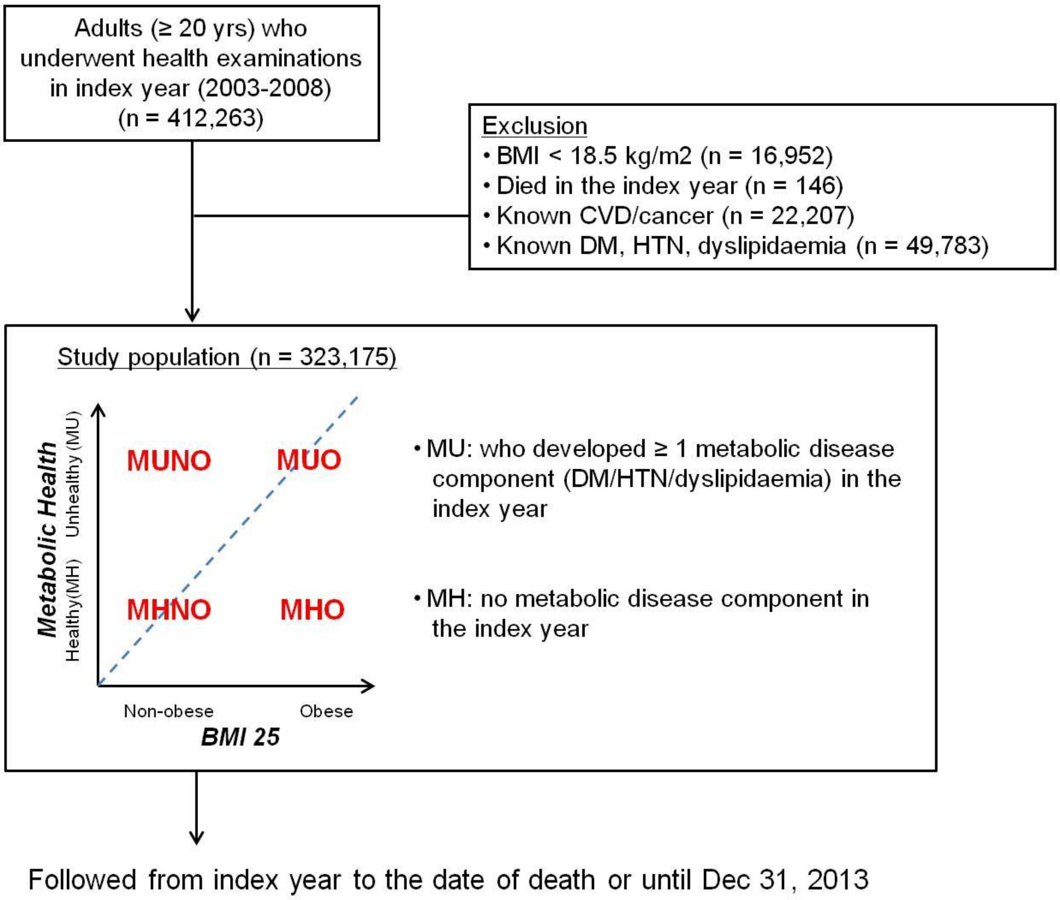

Supplementary Figure 2. Kaplan-Meier estimates of cumulative incidence of mortality

Abbreviations: CV, cardiovascular; MHNO, metabolically healthy non-obese; MHO, metabolically healthy obese; MUNO, metabolically unhealthy non-obese; MUO, metabolically unhealthy obese.

Survival curves were compared using the log-rank test. *$P < 0.001$ vs. MHNO, #$P < 0.05$ vs. MHNO.

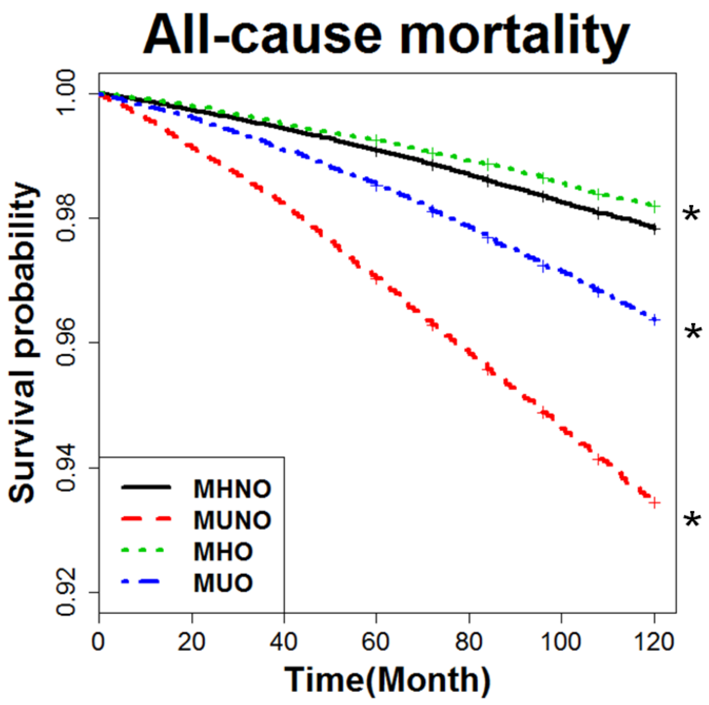

No. at risk

MHNO	172681	172233	171706	171129	128031	82498	45699
MUNO	56025	55556	55041	54395	43735	30850	17919
MHO	53303	53198	53041	52910	39508	25369	14207
MUO	41166	41011	40792	40576	32089	22620	13123

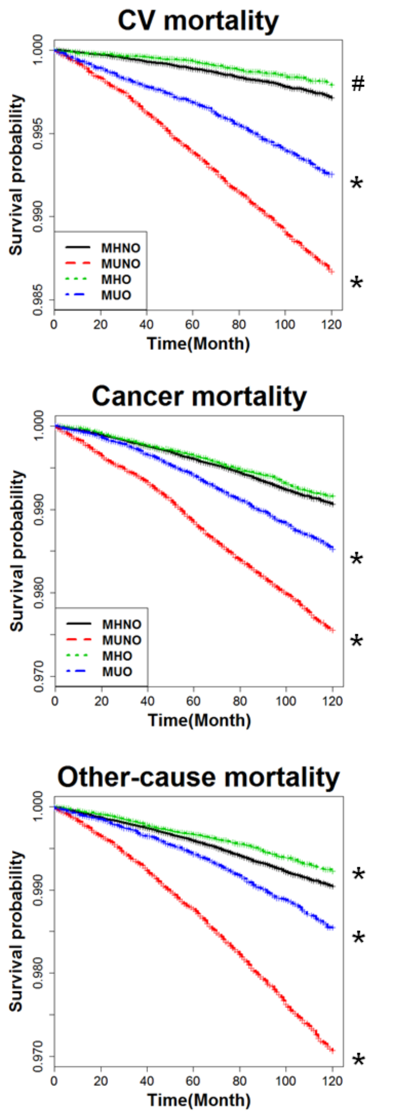

Supplementary Figure 3. Mortality according to BMI and metabolic health status

(Same analysis with Figure 1, but using BMI 25-30 kg/m^2 & metabolically healthy group as a reference.)

Abbreviations: CV, cardiovascular; MH, metabolically healthy; MU, metabolically unhealthy

The HRs (95% CI) were calculated using a Cox proportional hazards model and are adjusted for age, sex, smoking, alcohol drinking, exercise, and income status.

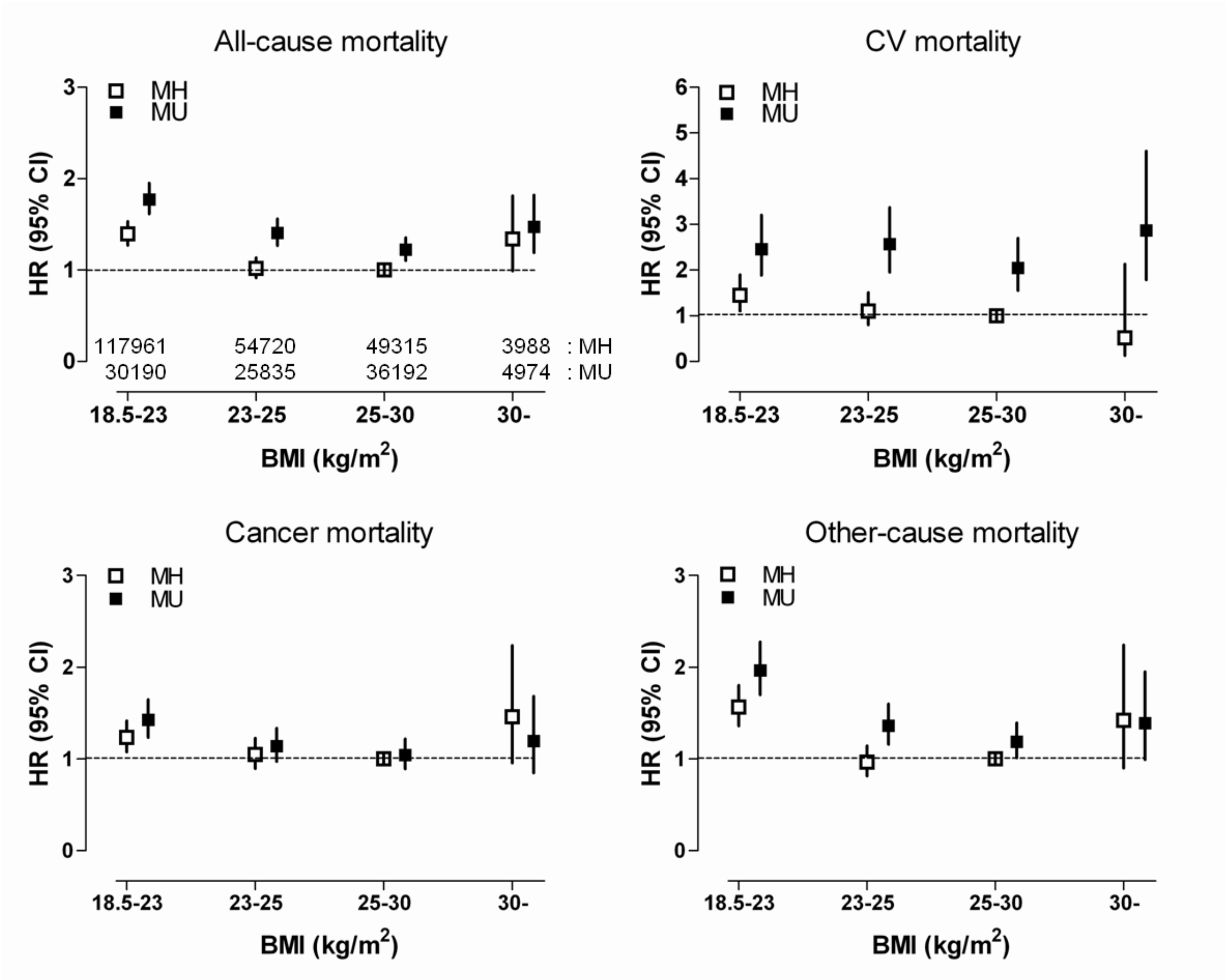

Supplementary Figure 4. Mortality according to the combination of metabolic diseases in non-obese (A) and obese (B) participants

Abbreviations: CV, cardiovascular; H, hypertension; D, diabetes; L, dyslipidaemia

The HRs (95% CI) were calculated using a Cox proportional hazards model and are adjusted for age, sex, smoking, alcohol drinking, exercise, and income status. A: non-obese (BMI < 25 kg/m^2) participants, B: obese (BMI ≥ 25 kg/m^2) participants.

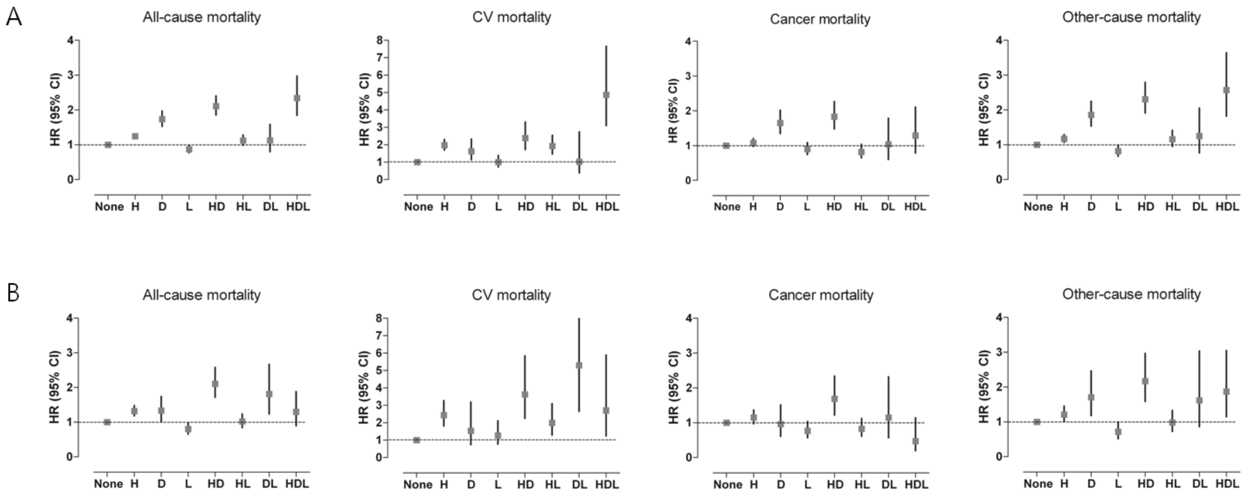

Supplement: Supplementary Information [file srep30329-s1.pdf]
